# Identification of hub genes and their correlation with infiltration of immune cells in *MYCN* positive neuroblastoma based on WGCNA and LASSO algorithm

**DOI:** 10.3389/fimmu.2022.1016683

**Published:** 2022-10-12

**Authors:** Ji Chen, Mengjiao Sun, Chuqin Chen, Bin Jiang, Yongjun Fang

**Affiliations:** ^1^ Department of General Surgery, Children’s Hospital of Nanjing Medical University, Nanjing, China; ^2^ Department of Hematology and Oncology, Children’s Hospital of Nanjing Medical University, Nanjing, China

**Keywords:** *MYCN*, neuroblastoma, WGCNA, LASSO, tumor microenvironment, immune cell infiltration

## Abstract

**Background:**

The prognosis of *MYCN* positive NB is poor, and there is no targeted drug for N-myc at present. This study aims to screen out hub genes closely related to *MYCN*, analyze the relationship between hub genes and NB microenvironment, and provide basis for molecular targeted therapy of *MYCN* positive NB.

**Methods:**

We combined the microarray data of GSE45547 (n=649) and GSE49710 (n=498), screened the DEGs between *MYCN* positive (n=185) and *MYCN* negative NB (n=951), performed WGCNA, Lasso regression and Roc analyses on the merged matrix, and obtained the hub genes related to *MYCN* in the training group. We performed ssGSEA on the experimental group to calculate the infiltration level of 28 kinds of immune cells in each sample, compared the differences of immune cell infiltration between *MYCN* positive and *MYCN* negative group. The influences of hub genes on the distribution of each immune cell were also analyzed by ssGSEA. The expression differences of the three hub genes were verified in the E-MTAB-8248 cohort (n=223), and the correlation between hub genes and prognosis of NB was calculated by Kaplan-Meier method in GSE62564 (n=498) and the validation group. We also verified the expression differences of hub genes by qRT-PCR in SK-N-BE(2), SKNDZ, Kelly and SH-SY5Y cell lines.

**Results:**

Here were 880 DEGs including 420 upregulated and 460 downregulated genes in *MYCN* positive NB in the training group. Overlap of the DEGs and WGCNA networks identified four shared genes, namely, *ZNF695*, *CHEK1*, *C15ORF42* and *EXO1*, as candidate hub genes in *MYCN* positive NB. Three core genes, *ZNF695*, *CHEK1* and *C15ORF42*, were finally identified by Lasso regression and Roc analyses. *ZNF695*, *CHEK1* and *C15ORF42* were highly expressed in *MYCN* positive NB tissues and cell lines. These three genes were closely related to the prognosis of children with NB. Except that Activated CD4 T cell and Type2 T helper cell increased, the infiltration levels of the other 26 cells decreased significantly in *MYCN* positive NB tissues. The infiltration levels of Type2 T helper cell and Activated CD4 T cell were also significantly positively correlated with the expression levels of the three hub genes.

**Conclusion:**

*ZNF695*, *CHEK1* and *C15ORF42* are highly expressed in *MYCN* positive NB, and their expression levels are negatively correlated with the prognosis of children with NB. The infiltration levels of Activated CD4 T cell and Type2 T helper cell increased in the microenvironment of *MYCN* positive NB and were significantly positively correlated with the expression levels of the three hub genes. The results of this study provide that *ZNF695*, *CHEK1* and *C15ORF42* may be potential prognostic markers and immunotherapy targets for *MYCN* positive NB.

## Introduction

Neuroblastoma (NB) is one of the most common solid extracranial malignancies in infants (1). Surgery combined with chemotherapy can achieve a good prognosis for children with low-risk (2) or intermediate-risk NB (3, 4). For high-risk NB, although combined surgery (5, 6), chemotherapy (5, 7), radiotherapy (5), immunotherapy (8–11), bone marrow transplantation (5) and other treatments, the prognoses of these patients remain poor (8). According to the revised NB risk classification system promulgated by Children’s Oncology Group in 2021, *MYCN* amplification is the most important biological factor affecting the risk staging of NB (12).

As an important member of *MYC* oncogene family, *MYCN* has highly conserved amino acid sequences with other members of *MYC* family, and these highly conserved amino acid sequences determine that members of *MYC* family have many identical biological properties (13). As an important transcription factor, N-myc protein formed by *MYCN* translation promotes the transcription of many genes related to NB metabolism (14), immune escape (15), apoptosis (16), ferroptosis (17) and other fields. Especially in the aspect of immune surveillance, recent studies have found that MYC family regulates the expression and production of a variety of immune ligands, receptors and immune effector molecules (18–20), thus affecting the biological activities of CD4^+^T (21), Macrophages (22), NK cells (23), B cells (24) and other cells, exerting an important influence on the microenvironment in NB (15). Although there have been attempts to use small molecules or low molecular weight compounds such as NY2267 (25), IIA6B17 (26), Tz-1 (27) and 10058-F4 (28) to target MYC family, no clinically available N-myc inhibitors can accomplish this task, and N-myc has long been thought to be an “untreatable” proto-oncoprotein (29). It has forced researchers to continue to innovate in targeted therapies for *MYCN*-driven malignancies.

Weighted gene Co-expression Network analysis (WGCNA) is a bioinformatics analysis method that is often used to effectively explore the relationship between genes and phenotypes (30). The significant advantage of WGCNA lies in its ability to aggregate genes into co-expression modules, bridging the gap between sample characteristics and gene expression changes. WGCNA analyzes thousands of genes to identify gene modules associated with clinical features, identifies key genes in disease pathways for further validation, and ultimately provides a system-level insight into signal networks that may be associated with a phenotype of interest.

**Figure 1:**
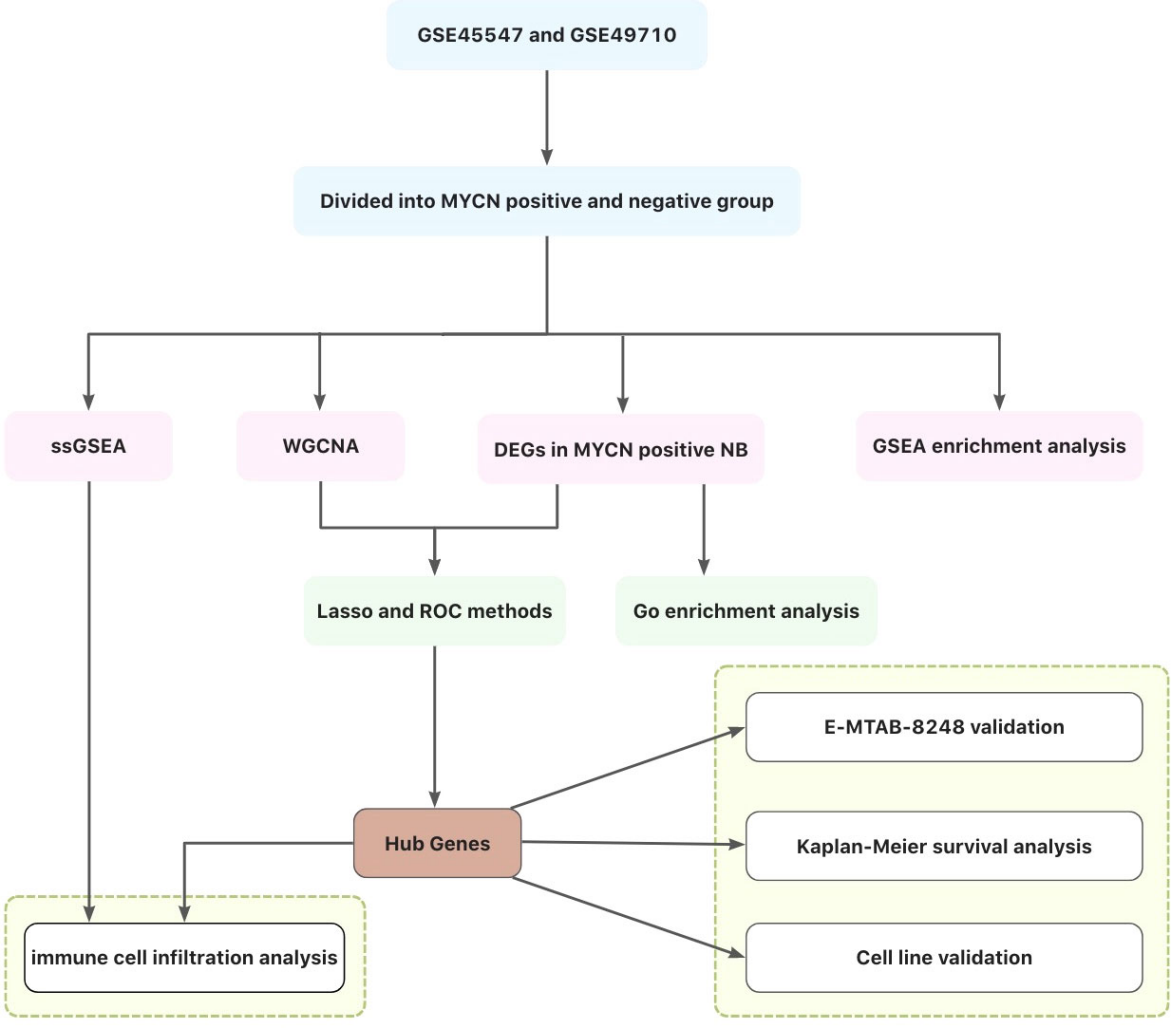
Flow chart of this study.

In this study, differentially expressed genes (DEGs) of *MYCN* positive NB were screened out by GEO and ArrayExpress databases, and gene co-expression network was constructed by WGCNA, Lasso regression and Roc analyses to obtain modules and hub genes that play important biological roles in *MYCN* positive NB. The immunocyte correlation analyses of *MYCN* positive NB, *MYCN* negative NB and hub genes were also carried out. **Figure 1** illustrates the workflow chart of data preparation, processing, analysis, and validation. The views provided in this paper will provide new insights into the immunotherapy of *MYCN* positive NB.

## Methods

### Cell culture

The NB cell lines used for the experiments including SK-N-BE (2), SKNDZ and SH-SY5Y were obtained from American Type Culture Collection (ATCC). Kelly cell line was kindly provided by Dr Guoliang Qing from Department of Pathophysiology, School of Basic Medical Sciences, Wuhan University. *MYCN* is highly expressed in SK-N-BE (2), SKNDZ and Kelly cell lines and low expressed in SH-SY5Y. All four cell lines are representative, widely used NB cell lines with relatively high feasibility. SK-N-BE (2), SKNDZ and SH-SY5Y cell lines were routinely cultured with DMEM (C11995500BT, Gibco, USA) supplemented with 10% fetal bovine serum (10099-141C, Gibco, USA) and1% Penicillin/Streptomycin (C100C5, New cell & Molecular Biotech, China), Kelly cell line was grown in complete RPMI-1640 (C11875500BT, Gibco, USA) supplemented with 10% FBS and 1% penicillin/streptomycin. All short tandem repeat (STR) -certified cell lines were cultured at 37C and 5% CO2 for up to 6 months after resuscitation, with mycoplasma contamination detected regularly using MycoAlert (LT07-710, Lonza, Switzerland).

### RNA extraction and real-time quantitative PCR (qRT-PCR)

Total RNAs were extracted from NB cells by Trizol reagent (15596018, ambion, USA). Then cDNA was synthesized using HiScript@IIQ RT SuperMix (R222-01, Vazyme, China) and qRT-PCR was performed with AceQ qPCR SYBR Green Master Mix (Low ROX Premixed)(Q131, Vazyme, China) according to the manufacturer’s instructions. The reaction conditions were: pre-denaturation at 95 C for 5 min, 40 cycles, denaturation at 95 C for 10 s, annealing at 60.0 C for 30 s, extension at 95C for 15 s, 60C for 60 s and 95 C for 15 s. qRT-qPCR was performed on a Lightcyler^@^ 96 instrument (05815916001, Roche, USA). Relative RNA amount was calculated by 2^-ΔΔCt^ (31)with the normalization to β-actin. β-actin is a cytoskeletal protein encoded by housekeeping gene, and its expression is relatively constant in various tissues and cell lines, so it is a suitable reference gene. The primer sequences (General Biol, China) were listed in **Table S1**.

### Data collection and DEGs analysis

Through data retrieval of GEO and ArrayExpress databases, GSE45547 (https://www.ncbi.nlm.nih.gov/geo/query/acc.cgi?acc=GSE45547), GSE49710 (https://www.ncbi.nlm.nih.gov/geo/query/acc.cgi?acc=GSE49710), E-MTAB-8248 (https://www.ebi.ac.uk/arrayexpress/experiments/E-MTAB-8248/) and GSE62564 (https://www.ncbi.nlm.nih.gov/geo/query/acc.cgi?acc=GSE62564) cohorts were selected as the objects of this research. The same patients were enrolled but different platforms were used in GSE62564 and GSE49710. All cohorts met the following criteria: 1) large sample size, 2) complete clinical information and microarray data, 3) fresh NB tissues for microarray analysis. Because of the same platform, GPL16876, GSE45547 and GSE49710 used, we integrated the data of the two cohorts into a large, fused expression matrix, after removing batch effects in order to obtain as many samples as possible to improve the statistic power. Meanwhile, E-MTAB-8248 cohort was selected as the validation group to fully verify the reliability of the results. GSE62564 was only used for survival analysis. After excluding the cases with unclear *MYCN* expression, 643 cases were included in GSE45547, including 93 *MYCN* positive cases and 550 *MYCN* negative cases. GSE49710 included 493 cases, including 92 *MYCN* positive cases and 401 *MYCN* negative cases. A total of 222 patients were included in the E-MTAB-8248 cohort, including 46 *MYCN* positive cases and 176 *MYCN* negative cases. Log2 transformation of transcriptome data was performed and probes were matched with genetic symbols according to the annotated documentation of the corresponding platform. Finally, the gene matrix with row name as sample name and column name as gene symbol was obtained for subsequent analysis. Data from GSE45547 and GSE49710 was averaged and combined into genomicMatrix as training group. By comparing the gene expression profiles of *MYCN* positive NB with those of *MYCN* negative NB, a list of DEGs with |log2fc|> 1.2 and p-value< 0.05 was obtained in the combined cohort.

### WGCNA

Data were preprocessed by “WGCNA” R package and abnormal genes were detected and excluded by the “GoodSamplegenes()” function. Then we used the “hclust()” function to draw the cluster graph, select the appropriate threshold, eliminate the outlier samples, and obtain relatively consistent gene expression data. We performed automatic network topology analysis according to the “pickSoftThreshold()” function, determined the soft threshold parameter (β) according to the principle of scale-free network and finally constructed a scale-free weighted gene co-expression network. Topological overlap measurement (ToM) was used to identify highly co-expressed gene modules to reduce the sensitivity of the network to false connections. By cutting the cluster tree into branches, the genes with high absolute correlation were clustered into the same module. Only modules with more than 60 genes could be defined as valid modules and the modules with high correlation would be merged (MEDissThres = 0.25). Each module would be assigned a different color for visual analysis. Cluster analysis of modules was based on the eigenvector value of each module. The “hclust()” and “merge()” functions were used to draw hierarchical clustering trees and merge modules with high correlation respectively. Finally, we plotted the scatter plot between Gene Significance (GS) and Module Membership (MM) within each module through the “plot()” function to understand the significance of high-connectivity genes within the module.

### Gene ontology (GO) enrichment analyses

GO function analysis was divided into three parts: biological process (BP), cell component (CC) and molecular function (MF). We performed GO enrichment analyses of the DEGs by “clusterProfiler” R package and drew bubble graph of GO enrichment analysis through “GGplot” R package.

### Gene set enrichment analysis

Gene set enrichment analysis (GSEA) was performed with “clusterProfiler” R package to calculate the gene sets with statistical differences between *MYCN* positive group and *MYCN* negative group. P-value < 0.05 was selected as the cutoff value. The annotated gene set entitled “immunesigdb” used in GSEA was downloaded from Molecular Signatures Database (MSigDB, https://www.gseamsigdb.org/).

### Establishment and validation of lasso regression analyses

The intersecting genes were obtained by overlap of DEGs (|log2fc|> 2.0 and p-value < 0.05) and hub genes, and the Lasso regression analysis was constructed by “Glmnet()” function. The minimum regularization parameter lambda (λ) and genes highly associated with *MYCN* positive NB were obtained by cross-validation (alpha=1). We draw receiver operator characteristic curve (Roc) for each gene by running “pROC()” function, calculated area under the curve (AUC) and evaluated the degree of association between *MYCN* positive NB and the hub genes in the training group and validation group. Survival curves of core genes were plotted by running the “SURv()” function, and Kaplan-Meier analysis was conducted to estimate the correlation between hub genes and prognosis.

### Analysis of immune cell infiltration

We performed single-sample gene set enrichment analysis (ssGSEA) on the training group using the “GSVA()” function to calculate the infiltration level of 28 kinds of immune cells in each sample, compared the difference of immune cell infiltration between *MYCN* positive and *MYCN* negative samples by Wilcoxon test and plotted the violin through the “Vioplot” R package. The correlation between 28 infiltrating immune cells and hub genes was further analyzed by “cor.test()” function, and the heatmap was drawn by “ggplot()” function.

## Results

### DEGs analysis

**Figure 2:**
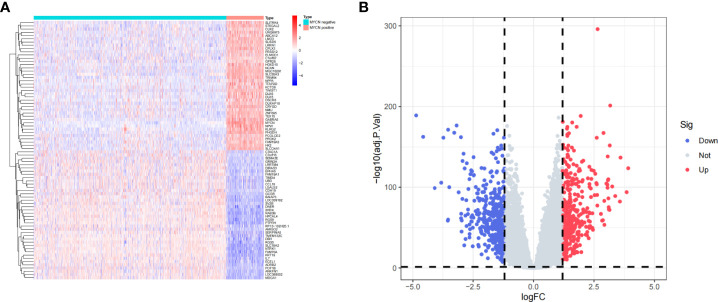
Identification of DEGs **(A)** Heatmap of representative DEGs in *MYCN* positive and *MYCN*-negative NB tissues. **(B)** Volcano plot of all genes in NB tissues. The vertical dotted lines represent |log2fc|= 1.2, the horizontal dotted lines represent p-value= 0.05.

A total of 1136 cases and 19806 genes were included by filtering and integrating the microarray data of GSE45547 and GSE49710 cohort. We selected 880 DEGs, including 420 highly expressed genes and 460 low expressed genes in *MYCN* positive NB by analyzing the gene expression levels of the two groups (|log2fc| > 1.2 and p-value < 0.05) (**Figures 2A, B** **)**. Among all DEGs, the 10 up-regulated genes with highest value of log2fc were *SLCO4A1*, *LMO3*, *MGC16291*, *ABCA12*, *HOXD10*, *TWIST1*, *NPW*, *GABRA5*, *NMU* and *DUXAP10*. The 10 down-regulated genes with minimal log2fc were *NTRK1*, *KRT19*, *LOC388002*, *IL7*, *RGS9*, *CCL19*, *RP13-102H20.1*, *SLC18A2*, *ECEL1* and *FAM70A*.

### GO enrichment analyses

**Figure 3:**
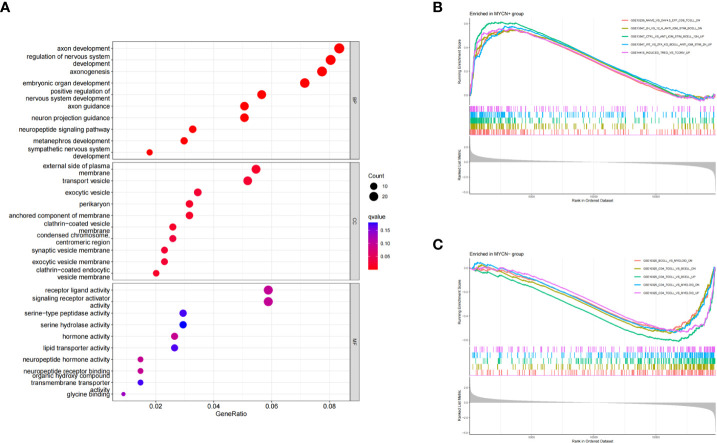
GO and GSEA enrichment analyses of DEGs **(A)** Bubble diagram of GO enrichment analysis of DEGs in *MYCN* positive NB. **(B)** GSEA analysis of representative immune-related pathways for all genes in *MYCN* positive NB. **(C)** GSEA analysis of representative immune-related pathways for all genes in *MYCN* negative NB.

Three subontologies of DEGs including biological process (BP), cellular component (CC) and molecular function (MF) were analyzed by GO analysis. 639, 43 and 20 pathways were enriched in each subontology respectively (**Figure 3A**). For BP, DEGs were mainly enriched in T cell activation (GeneRatio: 54/661, adjust p-value: 4.08×10^^-10^), T cell differentiation (GeneRatio: 36/661, adjust p-value: 3.78×10^^-9^), positive regulation of leukocyte cell-cell adhesion (GeneRatio: 32/661, adjust p-value: 1.48×10^^-7^), positive regulation of T cell activation (GeneRatio: 30/661, adjust p-value:1.73×10^^-7^) and lymphocyte differentiation (GeneRatio: 40/661, adjust p-value: 4.13×10^^-7^). With respect to CC, DEGs were mainly concentrated in MHC class II protein complex (GeneRatio: 10/684, adjust p-value: 4.01×10^^-11^), chromosome-centromeric region (GeneRatio: 27/684, adjust p-value: 1.53×10^^-9^), clathrin-coated endocytic vesicle membrane (GeneRatio: 16/684, adjust p-value: 2.81×10^^-9^), MHC protein complex (GeneRatio: 10/684, adjust p-value: 5.23×10^^-9^) and condensed chromosome, centromeric region (GeneRatio: 22/684, adjust p-value: 5.99×10^^-9^). DEGs were mainly clustered in MHC class II protein complex binding (GeneRatio: 10/664, adjust p-value: 1.72×10^^-8^), MHC protein complex binding (GeneRatio: 11/664, adjust p-value: 3.34×10^^-8^), hormone activity (GeneRatio: 18/664, adjust p-value: 3.95×10^^-7^), neuropeptide hormone activity (GeneRatio: 9/664, adjust p-value: 7.23×10^^-7^) and MHC class II receptor activity (GeneRatio: 5/664, adjust p-value: 1.32×10^^-5^) in MF category. These results suggested that a large number of *MYCN* related DEGs were enriched into immune-related pathways, and these DEGs might play important roles in the microenvironment of *MYCN* positive NB.

### GSEA

To explore the potential immune regulatory mechanism in NB, we used the annotated gene set entitled “immunesigdb” from MsigDB as a reference for GSEA analysis in this study. According to the standard of adjust p-value<0.05, A total of 439 immune-related pathways were enriched in *MYCN* positive NB (**Figure 3B**). The top five enrichment score pathways among *MYCN* positive NB were GSE15750_DAY6_VS_DAY10_TRAF6KO_EFF_CD8_TCELL_UP (enrichment score: 0.7155, NSE: 0.3534, adjust p-value: 3.12×10^^-9^), GSE15750_DAY6_VS_DAY10_EFF_CD8_TCELL_UP (enrichment score: 0.7082, NSE: 2.9948, adjust p-value: 3.12×10^^-9^), GSE36476_CTRL_VS_TSST_ACT_72H_MEMORY_CD4_TCELL_YOUNG_DN (enrichment score: 0.6930, NSE: 2.9574, adjust p-value: 3.12×10^^-9^) and GSE39556_CD8A_DC_VS_NK_CELL_MOUSE_3H_POST_POLYIC_INJ_UP (enrichment score: 0.6681, NSE: 2.8580, adjust p-value: 3.12×10^^-9^), GSE18893_TCONV_VS_TREG_24H_TNF_STIM_UP (enrichment score: 0.6648, NSE: 2.8257, adjust p-value: 3.12×10^^-9^). 1904 immune-related pathways were enriched in *MYCN* negative NB (**Figure 3C**). The top five pathways enriched among *MYCN* negative NB were GSE7218_UNSTIM_VS_ANTIGEN_STIM_THROUGH_IGG_BCELL_DN (enrichment score: -0.7259, NSE: -2.9380, adjust p-value: 3.12×10^^-9^), GSE7218_IGM_VS_IGG_SIGNAL_THGOUGH_ANTIGEN_BCELL_DN (enrichment score: -0.6682, NSE: -2.7121, adjust p-value: 3.12×10^^-9^), GSE10325_LUPUS_CD4_TCELL_VS_LUPUS_BCELL_UP (enrichment score: -0.6428, NSE: -2.6871, adjust p-value: 3.12×10^^-9^), GSE7509_UNSTIM_VS_FCGRIIB_STIM_DC_DN (enrichment score: -0.6405, NSE: -2.6303, adjust p-value: 3.12×10^^-9^) and GSE7509_UNSTIM_VS_IFNA_STIM_IMMATURE_DC_DN (enrichment score: -0.6363, NSE: -2.6296, adjust p-value: 3.12×10^^-9^). The above results fully indicated that there were significant differences in immune-related pathways enriched in *MYCN* positive and *MYCN* negative NB, especially in the expression of CD4 T, CD8T, B cell and other immune cells. *MYCN* and *MYCN* related genes might play an important role in the immune regulation of NB.

### Identification of clinically significant module and hub genes by WGCNA

**Figure 4:**
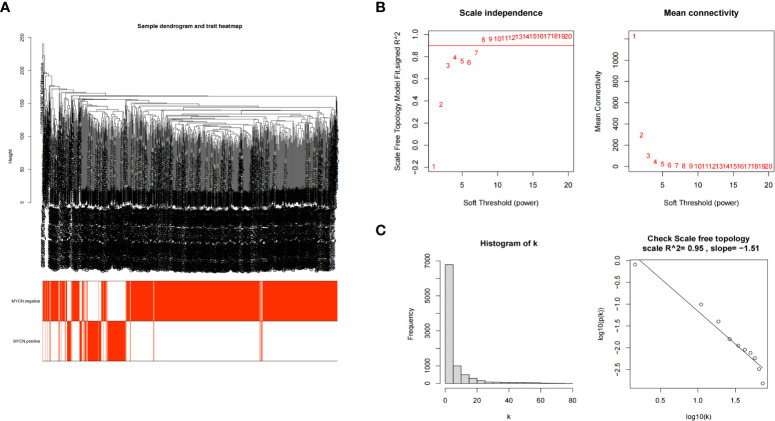
Establishment of scale-free network **(A)** Clustering dendrogram of 1136 samples. **(B)** Scale-free exponential analysis and average connectivity analysis of various soft-threshold powers (β). **(C)** Check the scale-free topology, the distribution approximately follows an approximate straight line, which is called approximate scale-free topology when β=8.

**Figure 5:**
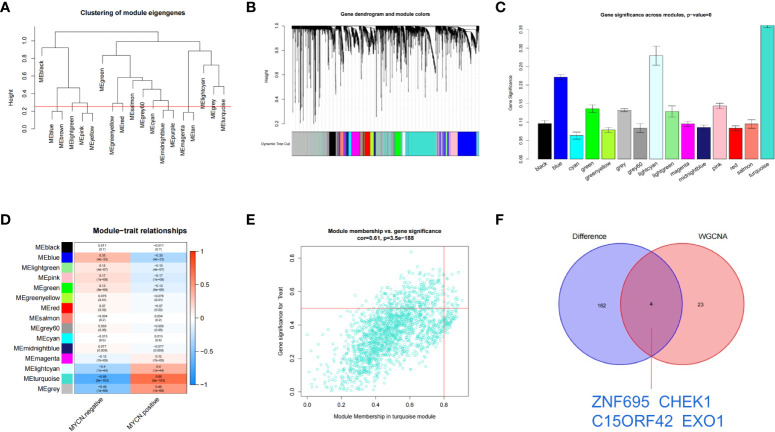
Identification of clinically significant module and hub genes by WGCNA **(A)** As shown in the hierarchical clustering tree, 19 modules were screened out and similar modules were further combined into 15 modules based on WGCNA (Red line: MEDissThres = 0.25). **(B)** Merged dynamic tree of genes with dissimilarity based on topological overlap and assigning module colors. **(C)** Diagram of correlation of NB and module’s color. The turquoise module was most closely related to *MYCN*. **(D)** Heatmap of the correlation between *MYCN* expression and module eigengenes. The turquoise module had the lowest p value and was selected as our next research object. **(E)** Scatterplot analysis of turquoise modules. core genes were filtered in the upper right region of GS > 0.5 and MM > 0.8. **(F)** Displays the Venn plot of 27 core genes in turquoise modules and 166 DEGs (|log2fc| > 2 and p-value < 0.05).

The transcriptome expression data of 185 *MYCN* positive and 951 *MYCN* negative NB tissues were preprocessed, and duplicated genes and missing values were removed to obtain a combined matrix containing 19806 genes. To ensure the accuracy of the results, we performed cluster analysis on the samples after removing the outlier samples. The sample dendrogram and trait heatmap are shown in **Figure 4A**. We selected the minimum threshold 8 that could make the curve tend to be smooth to make the co-representation network to be closed to a scale-free network (**Figures 4B, C** **)**. Nineteen modules were initially obtained and similar modules were further combined into 15 modules as shown in the hierarchical clustering tree (**Figures 5A, B** **)**. We found that turquoise module contained the largest number of genes among all modules (**Figure 5B**), with a total of 1842 genes included. The genetic importance of each module was listed in **Figure 5C**, from which we could see that turquoise module had the greatest effect on the gene regulatory network in NB (**Figure 5C**). In order to further understand the correlation between each module and *MYCN*, we analyzed the clinical information of training group and calculated the p-value of correlation between each module and *MYCN* expression level (**Figure 5D**), so as to screen out the module most related to *MYCN*. We could find that except the black module, salmon module and cyan module, all other modules were correlated with the expression level of *MYCN*. Among them, magenta module, lightcyan module, grey module and turquoise module were positively correlated with the amplification of *MYCN*, while other modules are negatively correlated with the expression of *MYCN*. Finally, turquoise module with the strongest correlation with *MYCN* (correlation index: 0.69, p-value: 5×10^^-163^) was obtained as the object of our further study. According to the filtering conditions of gene significance (GS)>0.5 and module membership (MM)>0.8, a total of 27 core genes were screened in turquoise module (**Figure 5E**). We crossed 166 DEGs (|log2fc| > 2 and p-value < 0.05) with 27 core genes of turquoise module and obtained four hub genes: *ZNF695* (GS: 0.6229 MM: 0.8559), *CHEK1* (GS: 0.5655 MM: 0.8746), *C15ORF42* (GS: 0.5479 MM: 0.9063), *EXO1* (GS: 0.5051 MM: 0.8569) (**Figure 5F**).These results suggested that there were multiple gene clusters in the gene network of NB. Due to the differential expression of *MYCN*, these gene clusters played different roles in NB. Exploring hub genes in turquoise module would help us understand the important roles of *MYCN* and its related genes in the gene regulatory network of NB.

### *MYCN* positive NB model constructed by Lasso regression and Roc analysis

**Figure 6:**
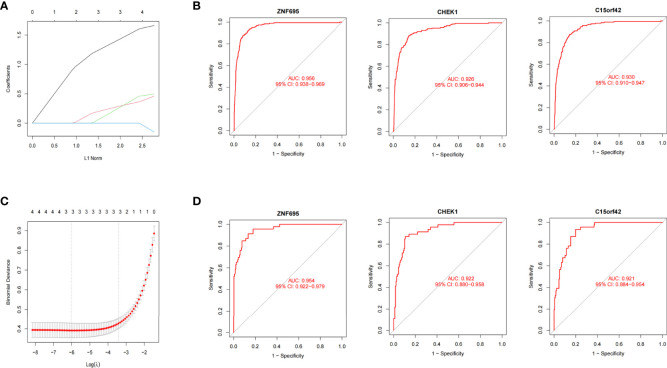
*MYCN* positive NB model constructed by Lasso and Roc regression analysis **(A)** Ten-fold cross-validation of tuning parameter selection in Lasso models. **(B)** Lasso coefficient curves of four *MYCN* closely related DEGs. *ZNF695*, *CHEK1* and *C15ORF42* were eventually screened out. **(C)** The Roc curves of the three hub genes in 1136 cases. **(D)** The Roc curves of the three hub genes in the validation set of E-MTAB-8248.

We further constructed Lasso regression model for the four genes to obtain the characteristic genes of *MYCN*. Three characteristic genes (*ZNF695*, *CHEK1*, *C15ORF42*) highly associated with *MYCN* positive NB were obtained by cross-validation (alpha=1) (**Figures 6A, B** **)**. Roc regression analysis was further performed to evaluate the association between the three genes and *MYCN* positive NB. In the training group, the AUC values of *ZNF695*, *CHEK1* and *C15ORF42* reached 0.956, 0.926 and 0.930 respectively (**Figure 6C**). In order to verify the results of the training group, we further calculated the AUC values of *ZNF695* (0.954), *CHEK1* (0.922) and *C15ORF42* (0.921) in the validation group (**Figure 6D**). These results indicated that *ZNF695*, *CHEK1* and *C15ORF42* are closely related to *MYCN* positive NB, which is worthy of further study.

### Validation of correlation between *ZNF695*, *CHEK1*, *C15ORF42* and *MYCN* positive NB

**Figure 7:**
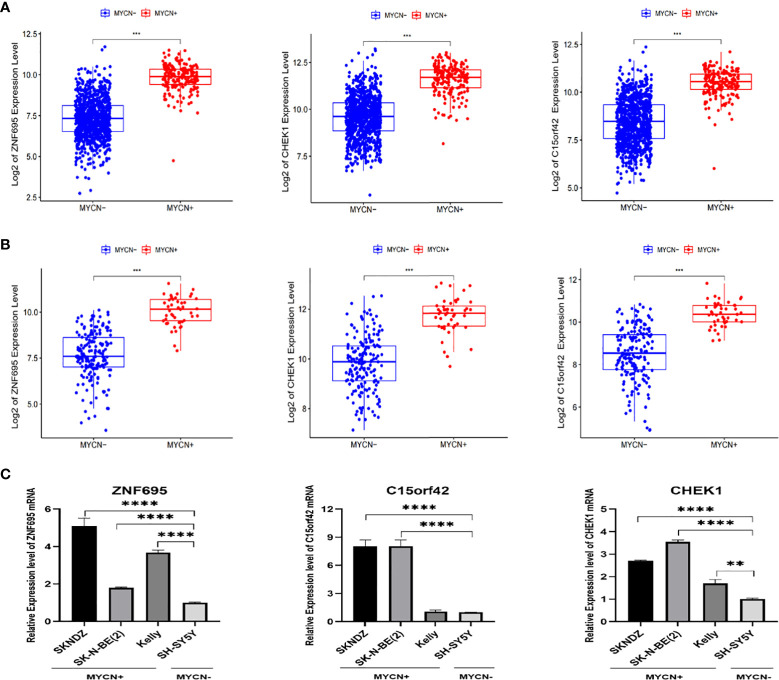
Validation of correlation between *ZNF695*, *CHEK1*, *C15ORF42* and *MYCN* positive NB **(A)** The comparison of the expression levels of the hub genes between the *MYCN* positive and negative group with a total of 1136 NB cases. **(B)** The comparison of the expression levels of the hub genes between the *MYCN* positive and negative group in validation set. **(C)** The comparison of the expression levels of the hub genes between the *MYCN* positive (SKNDZ, Kelly, SK-N-BE(2)) and negative (SH-SY5Y) cell lines. (**p<0.01, ***p<0.001, ****p<0.0001).

**Figure 8:**
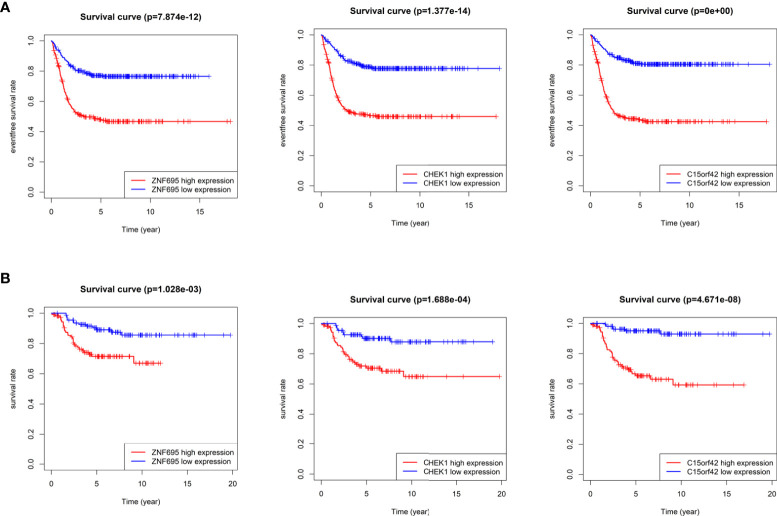
Survival analysis of three identified hub genes **(A)** Event-free survival analysis of three hub genes in GSE62564 cohort. **(B)** Overall survival analysis of three hub genes in E-MTAB-8248 cohort.

According to the boxplot, we found that *ZNF695*, *CHEK1* and *C15ORF42* were significantly overexpressed in *MYCN* positive NB in the training group (**Figure 7A**), and the same results were obtained in the verification group (**Figure 7B**). We also confirmed differential expression of *ZNF695*, *CHEK1*, and *C15ORF42* genes in *MYCN* positive NB cell lines SK-N-BE (2), Kelly, SKNDZ and *MYCN* negative cell line SH-SY5Y except for the expression of *C15ORF42* in Kelly cell line by qRT-PCR (**Figure 7C**). Survival analysis showed that *ZNF695*, *CHEK1* and *C15ORF42* had significant effects on event-free survival rate (GSE62564 cohort; **Figure 8A**) and overall survival rate (E-MTAB-8248 cohort; **Figure 8B**) in NB. These results indicated that the three hub genes screened in this study had highly consistent expression trends in NB tissues and cell lines, and their expression levels were closely related to the prognosis of children with NB. This provided bases for us to carry out researches on the mechanism of these three genes in NB.

### Correlation between immune cell status and *MYCN*-related DEGs in NB

**Figure 9:**
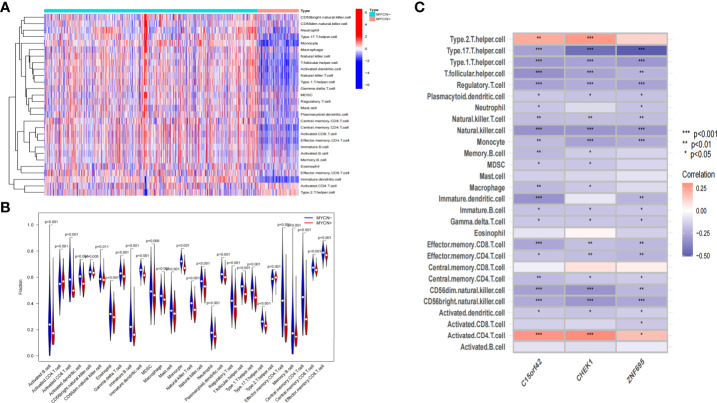
Correlation between immune-related cells and *MYCN*-related hub genes in NB **(A)** Heatmap shows the compositions of infiltrated immune cells between *MYCN* positive and *MYCN* negative group with a total of 1136 NB cases. **(B)** The violin plot shows the abundance of 28 immune-related cells in 1136 cases, comparisons between immune cells in *MYCN* positive and *MYCN* negative NB. **(C)** Heatmap shows the correlations between 28 immune-related cells and three hub genes in 1136 cases.

According to the microarray data, we further scored the immune cell infiltration status of 1136 NB cases in the experimental group, and a total of 28 immune-related cells were included in this study. As shown in the heatmap, the level of infiltration of immune cells was closely related to the expression level of *MYCN* (**Figure 9A**). It was found that the infiltration level of each immune cell showed an obvious opposite trend in *MYCN* positive and *MYCN* negative NB. Except that activated CD4 T cells and Type 2 T helper cells were significantly enriched in *MYCN* positive NB, the infiltration level of other immune cells showed an increasing trend in *MYCN* negative NB. In order to more intuitively reflect the distribution level of each immune cell in *MYCN* positive and *MYCN* negative NB, we drew the corresponding violin diagram, from which we could see that the distribution of 28 immune cells was different in *MYCN* positive and *MYCN* negative group (**Figure 9B**). Except that Activated CD4 T cell and Type2 T helper cells increased, the infiltration levels of the other 26 cells decreased significantly in *MYCN* positive NB tissues. This made us realize that immune cells might play an important role in *MYCN* positive NB, and the infiltration level of immune cells could also reflect the expression level of *MYCN*. Finally, we analyzed the correlation between the level of immune cell infiltration and three characteristic genes. The results showed that the infiltration levels of Type2 T helper cell and Activated CD4 T cell were significantly positively correlated with the expression levels of the three hub genes, while the infiltration levels of other 26 immune cells, especially Type17 T helper cell, Type1 T helper cell, T follicular helper cell, Regulatory T cell, Natural killer T cell, Natural killer cell, Monocyte, Immature dendritic cell, Effector memory CD8 T cell, CD56dim natural killer cell and CD56bright natural killer cell decreased significantly with the increased expression level of three hub genes (**Figure 9C**). These results suggested that *MYCN* and *MYCN* related genes have consistent effects on the immune cell infiltration in the NB microenvironment, and *MYCN* may regulate the microenvironment of *MYCN* positive NB partly through these hub genes.

## Discussion

Tumor formation is a very complex process, which involves the abnormal expression of a large number of tumor related genes and the changes of tumor microenvironment (32). It is particularly important to deeply understand the tumor microenvironment and strengthen the research of drugs targeting the tumor microenvironment. With the development of high-throughput sequencing (33) and single cell sequencing technology (34), the understanding level of tumor microenvironment has been further improved. Therefore, based on GEO and Arrayexpress database, this paper screened out the core genes closely related to *MYCN* positive NB and revealed the relationship between these genes and NB microenvironment.

We screened *MYCN* related genes and found that even if we raised the screening criteria to |log2fc| > 1.2 and p-value < 0.05, 880 DEGs were screened out. As an important transcription factor, N-myc plays an important role in the tumor biological characteristics of *MYCN* positive NB. As the results showed, the target genes of N-myc such as *Twist1* (35), *GLDC* (36), *TP53* (37) were significantly increased in *MYCN* positive NB. Therefore, *MYCN* gene may regulate the expression of key genes in the process of tumor metabolism (14), cycle (38), apoptosis (16), immunity (15) and so on through the highly expressed N-myc protein.

By immune-related pathway enrichment of DEGs, we found that DEGs were significantly enriched in NB microenvironment, especially in T cells’ activation, differentiation, adhesion and formation of MHC proteins. GSEA enrichment analysis also showed that the regulation of CD8^+^T cells, CD4^+^T cells, NK cells and Treg cells was closely related to the expression level of *MYCN*. In the past few years, more attention has been paid to the research on MYC, there have also been studies focusing on the effect of age of onset on immune cell infiltration in NB (39). However, with a deeper understanding of *MYCN*, we have found that *MYCN* plays an important role in many fields of tumor microenvironment. For example, in the early years, we found that N-myc suppressed the expression of MHC I through enhancer inactivation (40). Subsequently, it was found that N-myc could mediate tumor immune escape by inhibiting NKT cell enrichment to the site of disease in NB (15). In recent years, it was found that N-myc could regulate the expression of PD-L1, thereby regulating tumor immune surveillance and immune escape (41). Through WGCNA and Lasso regression analysis, we finally screened three hub genes closely related to *MYCN*: *ZNF695*, *CHEK1* and *C15ORF42*. Since *MYCN* is closely related to NB tumor immunity, the roles of these three genes in the regulation of the microenvironment of *MYCN* positive NB are worthy of further study.

At present, there are few studies on *ZNF695*, let alone related reports on *ZNF695* in NB. According to the projections provided by Alliance of Genome Resources, *ZNF695* may enable DNA-binding transcription factor activity, RNA polymerase II-specific and RNA polymerase II cis-regulatory region sequence-specific DNA binding activity, and is predicted to be involved in regulation of transcription (https://www.alliancegenome.org/gene/HGNC:30954#summary). Ke ZB et al. found that *ZNF695* is an important marker of radiotherapy resistance in prostate cancer (42), and is closely related to the proliferation, invasion and migration of prostate cancer cells, which may be an important factor affecting the microenvironment of prostate cancer (42, 43).Takahashi T et al. found that methylation of *ZNF695* is an important factor affecting the chemotherapeutic resistance of esophageal squamous cell carcinoma (44). Some studies have also found that *ZNF695* is closely related to the prognosis of breast cancer (45) and ovarian cancer (46). These results fully indicated that *ZNF695* is closely related to the prognosis of many tumors, and may have an important role in the microenvironment of malignant tumors.

*CHK1* (*CHEK1*), a member of the *CHEK* family, is a serine/threonine-specific protein kinase that mediates cell cycle arrest in response to DNA damage (47). Previously, *CHK1* was considered to be a tumor suppressor gene because of its role in regulating DNA damage (48). However, current studies have found that this gene is overexpressed in some solid tumors and affects the prognosis of these patients (49). Therefore, it is of great significance to study the regulatory mechanism of *CHK1* in solid tumors. Previous studies have found that N-myc in *MYCN* positive tumor cells abnormally regulates G1/S checkpoint, inhibits G1 arrest after DNA damage, promotes tumor cells to enter the S phase with DNA damage repairing and experiencing chronic replication stress (50, 51). They are more dependent on ATR/CHK1 pathway to solve DNA damage during replication (51). *CHK1* inhibitors can well inhibit the protective effect of ATR/CHK1 pathway on *MYCN* positive tumor cells (52). A number of studies have shown that *CHK1* inhibitors or combined with Wee1 (53), R9-capep (54) and other drugs have significant killing effects on *MYCN* positive NB cell lines, and *CHK1* inhibitors can induces regression of preclinical models of human NB (55, 56). However, there are currently no reports on the correlation between *CHK1* and the microenvironment of *MYCN* positive NB.

**Figure 10:**
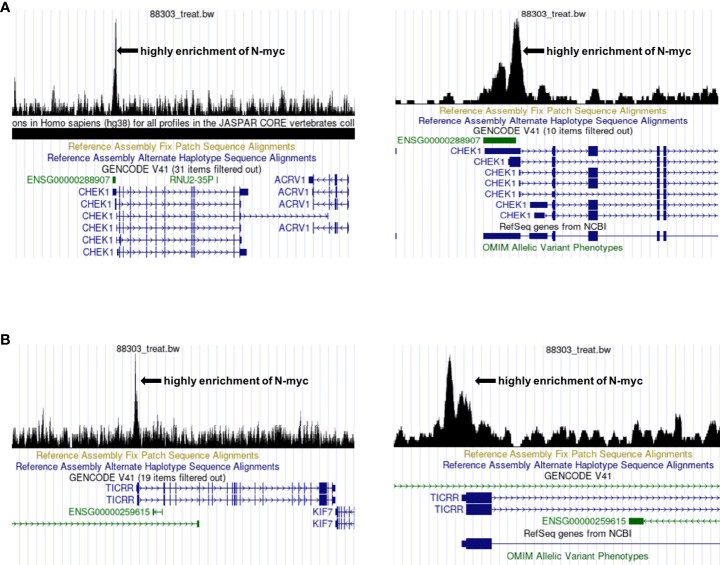
Prediction of the correlation between *CHEK1*, *C15ORF42* and *MYCN* **(A)** The UCSC database predicts that N-myc is highly enriched near the *CHEK1* transcription start site. **(B)** The UCSC database predicts that N-myc is highly enriched near the *C15ORF42* transcription start site.

*C15ORF42* (*TICRR*), a human homolog of yeast sld3 protein (57), is an important DNA replication factor regulated by cyclin dependent kinases and DNA damage checkpoints. Yu Q et al. suggested that *C15ORF42* participates in tumorigenesis by accelerating DNA replication, and a higher level of *C15ORF42* is associated with poor overall survival and disease free survival in multiple cancer types (58). For example, the increased expression of *C15ORF42* in papillary renal cell carcinoma may participate in tumorigenesis by regulating the cell cycle, and have an impact on prognosis (59). Wang Y et al. found that *C15ORF42* is closely related to the prognosis of pediatric brain tumors, and may affect the prognosis of children by changing the tumor microenvironment (60). At present, there are no studies on the effects of *C15ORF42* in the microenvironment of children with NB, and no researches on the correlation between *MYCN* and *C15ORF42*. We found that there are specific binding sites of N-myc and N-myc was highly enriched in the transcription initiation region of *CHK1* and *C15ORF42* through UCSC database (http://genome.ucsc.edu/) and chip-seq data from Zeid R’s research (61)**(** **Figures 10A, B** **)**, which suggests that N-myc may be an important transcription factor of *CHK1* and *C15ORF42*.

In conclusion, the precise mechanisms of *ZNF695*, *CHEK1* and *C15ORF42* in *MYCN* positive NB are still unclear. This study successfully predicts that these three genes, as key genes in the regulation of tumor biological characteristics in *MYCN* positive NB, are closely related to tumor microenvironment. The limitation of this study is that all the above results are based on microarray data and bioinformatics technology, and have not been fully verified by experiments. In the next step, we will further explore the mechanism of the correlation between these three hub genes and the microenvironment of *MYCN* positive NB.

## Conclusion

The treatment of *MYCN* positive high-risk NB is still a difficulty in clinical work, and currently there is no targeted drug directly targeting N-myc. In this study, three hub genes closely related to *MYCN* were screened out by WGCNA and Lasso regression analysis through GEO and ArrayExpress databases. It is predicted that these three genes may be closely related to the microenvironment of *MYCN* positive NB. Subsequent mechanism studies may bring new ideas for the clinical diagnosis and treatment of *MYCN* positive NB and bring hope to children with NB.

## Data availability statement

The datasets presented in this study can be found in online repositories. The names of the repository/repositories and accession number(s) can be found in the article/**Supplementary Material**.

## Author contributions

BJ and YF, conceptualization, methodology, writing-reviewing, and editing. JC and MS, investigation, data curation, and writing-original draft preparation. JC and CC, visualization, validation, supervision, and software. All authors contributed to the article and approved the submitted version.

## Funding

This study was supported by the General Project of Nanjing Medical University (NMUB20210060), National Natural Science Foundation of China (81903383), Natural Science Foundation of Jiangsu Province (BK20211009), Scientific Research Projects of Jiangsu Health Commission (ZDB2020018), China Postdoctoral Science Foundation funded project (2021M701764), Special Fund for Health Science and Technology Development in Nanjing (JQX19008), Nanjing Medical Science and Technology Development Project (YKK21149), Young Talent Support Project of Children’s Hospital of Nanjing Medical University (TJGC2020016, TJGC2020007, TJGC2020014).

## Conflict of interest

The authors declare that the research was conducted in the absence of any commercial or financial relationships that could be construed as a potential conflict of interest.

## Publisher’s note

All claims expressed in this article are solely those of the authors and do not necessarily represent those of their affiliated organizations, or those of the publisher, the editors and the reviewers. Any product that may be evaluated in this article, or claim that may be made by its manufacturer, is not guaranteed or endorsed by the publisher.
