# Supplementary material for: Identification of hub genes and their correlation with infiltration of immune cells in MYCN positive neuroblastoma based on WGCNA and LASSO algorithm
Source: Front Immunol. 2022 Oct 12;13:1016683. doi: 10.3389/fimmu.2022.1016683 (PMC9596756; doi:10.3389/fimmu.2022.1016683)
Supplement: Supplementary file 1 [file Table_1.docx]

| **Table S1: The sequences of primers for qRT-PCR** | | |
| --- | --- | --- |
| **Name** |  | **Sequence** |
| ZNF695 | Forward | 5’- ATCTCCCTTGGTGAGGATAGC -3’ |
|  | Reverse | 5’- GACAAAACTGAGTGTTTGGCTG -3’ |
| CHEK1 | Forward | 5’ - GACAAAACTGAGTGTTTGGCTG -3’ |
|  | Reverse | 5’- TGCCTATGTCTGGCTCTATTCTG -3’ |
| C15ORF42 | Forward | 5’- CCTCACCTATCTGAGTTGCCG -3’ |
|  | Reverse | 5’- CACTGGTAGTCTAGCAGCGT -3’ |
| β-actin | Forward | 5’- CATGTACGTTGCTATCCAGGC -3’ |
|  | Reverse | 5’- CTCCTTAATGTCACGCACGAT -3’ |
